# In reply: 

**DOI:** 10.3325/cmj.2021.62.305

**Published:** 2021-06

**Authors:** Nada Starčević Čizmarević, Marin Tota, Smiljana Ristić

**Affiliations:** 1 Department of Medical Biology and Genetics, Faculty of Medicine, University of Rijeka, Rijeka, Croatia *nadasc@medri.uniri.hr*; 2 Department of Medical Chemistry, Biochemistry and Clinical Chemistry, Faculty of Medicine, University of Rijeka, Rijeka Croatia

We thank Dr Saadat (1) for his comment on our study. Dr Saadat extended the analysis of the relationship between COVID-19 prevalence/mortality and CCR5-Δ32 to 82 countries worldwide. The author reported a positive correlation between CCR5-Δ32 allelic frequency and square root-transformed (SR) prevalence (partial r = 0.265, *P* = 0.017) and mortality (partial r = 0.216, *P* = 0.053). Previously, we found no correlation between CCR5-Δ32 mutation frequency with log prevalence (partial r = -0.004, *P* = 0.979) and log mortality (partial r = -0.156, *P* = 0.355) of COVID-19 in 39 European countries (2). We believe that different results of these reports can be explained in several ways. The first explanation relates to the ethnicities/countries involved in the analyses. Our study was limited to Europe, where the populations share a common ancestry and do not largely differ in other risk covariates for COVID -19.

Saadat examined the impact of the CCR5-Δ32 mutation on global prevalence/mortality, including European/Asian/African countries, which differ significantly in genetic background, risk parameters that may influence the COVID-19 pandemic, and the CCR5 polymorphism prevalence. Indeed, the distributions of the CCR5 polymorphism are known to differ in European, Asian, and African populations (3). While CCR5-Δ32 allele frequency averages 10% in European populations, it is rare or absent in Asians and black Africans.

A second possible explanation is the timing of the analyses. We analyzed data evaluated on June 1, 2020, during the first wave of COVID-19 (when social restrictions were imposed in all European countries) and Saadat analyzed the data obtained from late December 2020, during the second pandemic wave. Although each country had its own strategy in imposing and relaxing restrictions, European countries had a more consistent strategy compared with countries on other continents. During the first wave, European countries were largely in total lockdown, and when they opened their borders in late June 2020, social contact and viral transmission increased, leading to a second pandemic wave in early autumn. Therefore, it is desirable to conduct studies in countries that not only have a homogeneous genetic background but that also used a similar strategy to combat the COVID-19 pandemic.

Finally, the variability in results may also stem from differences in the approach to data analysis, such as adjustment for possible confounders. In both studies, the number of diagnostic tests performed (per 10^6^ individuals) was used as a potential confounder. In Saadat's report, the Human Development Index (HDI) was also used as a potential confounder, which is a very valuable strategy considering that HDI values differ significantly between the countries included in the study. HDI values do not differ much between European countries, and when we subsequently included HDI as a potential confounder, our results remained unchanged. On the other hand, we used the time elapsed (days since January 1, 2020) since the outbreak of the epidemic in each country as a potential confounder, taking into account that the timing of the epidemic outbreak differed in different countries/continents. For example, the SARS-CoV-2 outbreak in North Africa and Middle East was significantly delayed compared with the outbreak in Asia and Europe.

From all this, it can be concluded that it would be desirable to analyze populations that are as homogeneous as possible and compare the results for European/Asian/African countries. Such population-based studies could have an important clinical implication for the detection and management of COVID-19 and promote future research on this topic.

## References

[1] Saadat M The prevalence and mortality of COVID-19 associated with the allelic frequency of CCR5-Δ32. Croat Med J 2020;62:303-304

[2] Starčević Čizmarević N, Tota M, Ristić S (2020). Does the CCR5-Δ32 mutation explain the variable coronavirus-2019 pandemic statistics in Europe?. Croat Med J.

[3] Solloch UV, Lang K, Lange V, Böhme I, Schmidt AH, Sauter J (2017). Frequencies of gene variant CCR5-Δ32 in 87 countries based on next-generation sequencing of 1.3 million individuals sampled from 3 national DKMS donor centers.. Hum Immunol.

